# Initial Antihypertensive Prescription and Switching: A 5 Year Cohort Study from 250,851 Patients

**DOI:** 10.1371/journal.pone.0053625

**Published:** 2013-01-14

**Authors:** Martin C. S. Wong, Wilson W. S. Tam, Clement S. K. Cheung, Ellen L. H. Tong, Antonio C. H. Sek, George John, N. T. Cheung, Bryan P. Y. Yan, C. M. Yu, Stephen Leeder, Sian Griffiths

**Affiliations:** 1 School of Public Health and Primary Care, Faculty of Medicine, Chinese University of Hong Kong, Hong Kong; 2 Hospital Authority Information Technology Services, Health Informatics Section, Hong Kong; 3 University of Oxford, Oxford, United Kingdom; 4 Department of Medicine and Therapeutics, Faculty of Medicine, Chinese University of Hong Kong, Hong Kong; 5 Menzies Centre for Health Policy, University of Sydney, Sydney, Australia; Thomas Jefferson University, United States of America

## Abstract

**Purpose:**

Adverse effects of antihypertensive therapy incur substantial cost. We evaluated whether any major classes of antihypertensive drugs were significantly associated with switching as a proxy measure of medication side effects in a large Chinese population in Hong Kong.

**Methods:**

From a clinical database, all adult patients newly prescribed an antihypertensive mono-therapy in Hong Kong between the years 2001–2003 and 2005 were included. Those who paid only one visit, died or stayed in the cohort for <180 days after the prescription, or prescribed more than one antihypertensive agent were excluded. The factors associated with switching at 180 days were evaluated by multivariate regression analyses. Age, gender, payment status, service type, district of residence, drug class, systolic and diastolic blood pressure levels were predictor variables.

**Results:**

From 250,851 subjects, 159,813 patients were eligible. A total of 6,163 (3.9%) switched their medications within 180 days. Patients prescribed thiazide diuretics had the highest switching rate (5.6%), followed by ACEIs (4.5%), CCBs (4.4%) and beta-blockers (3.2%). When compared with ACEIs, patients on thiazide diuretics were significantly more likely to be switchers (adjusted odds ratio [AOR] 1.49, 95% C.I. 1.31–1.69, p<0.001), whilst patients prescribed CCBs and beta-blockers were similarly likely to have switching. Following these patients up for 5 years showed that thiazide had the most marked increase in switching rate.

**Conclusions:**

The higher rates of switching among thiazide diuretics in this study might raise a probably greater incidence of their adverse effects in this Chinese population, yet other factors might also influence switching rates. Patients prescribed thiazide diuretics for longer term should be observed for their intolerability.

## Introduction

Hypertension is the biggest contributor to global mortality and is rising in prevalence [1], [2]. It affects more than 65 million adult Americans, and data from the National Health and Nutrition Examination Survey 1999–2000 showed that 28.7% of people had hypertension [3], [4]. A recent study among 25,196 urban adults aged 18–74 years in northeast of China showed the prevalence of elevated blood pressure was 40.5% [5], which conferred increased cardiovascular risks [6]–[7]. In addition, hypertension has also been evaluated as the most contributory component of metabolic syndrome in the association with ischemic heart disease in Taiwanese Type 2 Diabetic Patients [8]. Despite clinical consequences of uncontrolled hypertension as expressed in morbidity and mortality from cardiovascular disease and stroke being well recognized [2], [9], [10], very small proportions of patients achieve control at less than 140/90 mmHg [11]. This problem is worse in countries belonging to the Asia Pacific region like China, where recent studies have found that only 3.5% of rural and 3.7% of urban hypertensive patients achieved optimal control [12], [13]. It has been estimated by the American Heart Association that the yearly direct and indirect costs of hypertension exceeded 93.5 billion in 2011, and together with stroke and cardiovascular diseases, accounts for 17% of the total healthcare expenditure per year in the US [14].

A substantial body of evidence has shown that antihypertensive medications could reduce these complications [15]. However, not all patients on pharmacotherapy receive the full potential benefit from treatment. Adverse effects of drugs or inefficacy of blood pressure control have been one major causes of switching of antihypertensive agents in clinical practice [16].

Therefore, the profiles of drug therapy, especially the history of switching medications among the major antihypertensive drug classes provide important information for physicians during clinical consultations, especially in the shared decision making process when a first-ever antihypertensive drug is prescribed. Most previous studies regarding this important problem have been conducted among Caucasian patients [17]–[19] and only a few large-scale studies have been done in the Chinese population. As a result, we are uncertain whether these studies can be generalized to Chinese ethnic origin, especially when ethnicity has been documented as influencing the pharmacological action of antihypertensive agents [20].

We have previously reported the rates of switching among antihypertensive agents in Chinese adults. Age, gender, visit type (new clinic visitors vs. follow-up consultation), number of comorbidities and socioeconomic status [21]–[24] were significant independent factors determining switching. Thiazide diuretics were found to be significantly more likely to be switched 180 days after their prescriptions when compared with other antihypertensive drug classes [22]. However, these studies were not exclusively patients who could be considered members of inception cohorts, as patients who had already taken antihypertensive agents were included, and the study was restricted to one of seven regions of Hong Kong. Moreover, no blood pressure levels were available as a covariate in regression analyses, and we included patients who were all seen in public primary care settings. In addition, the follow-up period was restricted to a short period of 180 days. The objective of this study was to evaluate the longer-term switching rates according to drug classes among all Hong Kong residents, and it tested the *a priori* hypothesis that there existed differences in switching rates among the major antihypertensive drug classes as reported in our previous study [22].

## Materials and Methods

This study was approved by the “Clinical Research Ethical Committee, the Hospital Authority, Hong Kong”, and “the Survey and Behavioural Research Ethics Committee, Faculty of Medicine, Chinese University of Hong Kong”. Informed consent was waived as we anonymized all patient records and replaced patient names with a unique identifier.

### Source of Data

This study utilized the Clinical Data Analysis Reporting System (CDARS) of the Hospital Authority (HA) of Hong Kong, which consists of seven million patient records, one million annual admissions and 13 million ambulatory visits. One of its objectives is to provide data for research [25]. This database contained clinical and demographic information of patients, clinical diagnoses in terms of International Classification of Primary Care (ICPC)-2 or International Classification of Disease (ICD)-10 codes, types of clinical services (in-patient; specialist out-patient; general out-patient emergency department; and others), as well as drug prescription details. We have previously evaluated a similar database and reported a high level of data completeness on demographic (100%) and prescription details (99.98%) [26].

The HA provides free or low-cost primary and secondary care services to the public. When patients visit these hospital or clinics, they register at first attendance with their identity documents. Demographic and socioeconomic data is then entered into the computer system by clinic administrative staff in the reception office. After each consultation, physicians enter drug prescription details into the computer system, which is then double checked by at least two independent dispensers or pharmacists to assure correct drug dispensing. Prescriptions can only be issued via the computerized system. Any hand-written amendments to drug prescriptions by physicians and those requiring the patients to purchase in community pharmacies are also entered into the computer system. These computerized records are the only conduits for information entry by physicians at every patient visit in all primary care clinics run by the HA. Clinical guidelines are provided to all physicians regarding the need to enter the diagnosis of the consultation in the form of ICPC-2 or ICD-10 codes. Patients receiving public assistance are almost exclusively residents supported by social security allowances from the government. To apply for a fee waiver, patients are comprehensively assessed by medical social workers who determine their inability to pay medical consultation fees. Each consultation costs US$5.77 including investigation and prescription fees unless a fee waiver applies.

The data used in this study includes information from all patients in the whole Territory of Hong Kong. Hong Kong is divided into three geographically distinct regions - the New Territories, Kowloon and Hong Kong Island - from the most rural to the most urbanized. This translates to a population of over 7 million, a median age of 39 years, roughly equal number of males and females, and a median household income of US$2,240 [27], [28].

### Definition of the Cohort

We included all adult patients aged 18 years or older who attended any public healthcare services in Hong Kong during the study period from January 2001 to December 2005, and those who were prescribed an antihypertensive agent and attended a consecutive visit for antihypertensive drug refill. Patients who started medications in 2004 were not included in our analysis as the Severe Acute Respiratory Syndrome epidemic heavily affected the public healthcare system, limiting medication refill in that year. Those who died within 180 days of receiving their first prescription or stayed in the cohort for less than 180 days were excluded. We further classified each patient according to the presence of comorbid conditions that could influence the prescription choice of antihypertensive agents, including diabetes, impaired glucose tolerance, dyslipidemia, asthma, chronic lung disorders, cardiovascular diseases, stroke, and renal impairment.

Automated pre-calibrated blood pressure machines were used to measure blood pressure in patients attending the public health care system. Upon each clinic visit, patients’ Blood Pressure (BP) were measured once and if found elevated, it is usual practice to repeat the measurement after at least 5 minutes of rest, where the second reading was usually used as the clinic BP. From guidelines the diagnosis of hypertension was established by attending physicians if the patient had at least three separate readings of BP≥140/90 mmHg in different visits.

### Exposure to Drugs and Assessment of Switching

The major outcome variable in this study was switching from one antihypertensive medication to another within 180 days of prescription. Drug switching is defined as the absence of a refill prescription in all subsequent clinic visits combined with the prescription of another antihypertensive drug of a different class within 180 days since the date of the first prescription.

This definition is similar to previous studies [18], [29] except that a longer time (180 days) has been used instead of 90 days, taking into account the practice of prescribing antihypertensive drugs for a period of more than 90 days (range 14 to 168 days; median 56 days) among a significant proportion of physicians as found in our database.

### Covariables and Statistical Analysis

The drug switching group and non switching group were compared on the basis of sociodemographic variables. The classes of antihypertensive drugs prescribed included thiazide diuretics, beta-blockers, alpha-blockers, calcium channel blockers [CCB], angiotensin converting enzyme inhibitors [ACEIs], and combination therapy. Combination therapy was defined as prescriptions containing antihypertensive classes from at least two different drug classes.

A binary logistic regression analysis was conducted of all patients with the cumulative incidence of drug switching within 180 days as the outcome variable. We also followed all patients for 5 years and the switching rates were compared according to drug class one-yearly for 5 years. Potentially independent factors controlled for included age, gender, payment status (public assistance vs. fee payers), service type (in-hospital; specialist out-patients; Accident and Emergency Department; general out-patients; others), the districts of residence (Hong Kong Island, Kowloon and the New Territories), the presence of comorbidities, systolic blood pressure (SBP) and diastolic blood pressure (DBP) levels. The average of all blood pressure values entered into the computer over all clinic visits was used. The selection of independent variables was hypothesis-driven. All predictor variables were entered unconditionally into the regression equations. All covariates were tested for the absence of multi-collinearity. As part of the sensitivity analysis, we repeated the regression models with both SBP and DBP levels, SBP alone, DBP alone, and none. In addition, since the initial prescription of antihypertensive agents was not randomized, we used the inverse estimate propensity score for each drug class and included the variable into the regression analysis to detect any differences for the associated factors. We used the Statistical Package for Social Sciences (SPSS Incorporation, Chicago Illinois) version 16.0. All p values less than 0.05 were regarded as statistically significant.

## Results

### Patient Characteristics

In this large cohort of 223,287 patients receiving a newly-initiated antihypertensive agent, 27,238 visited the clinic setting only once, 4,391 were prescribed more than one antihypertensive agents, 31,845 stayed in the cohort for less than 180 days. These included patients who died within the first 180 days or were lost to follow-up. The total eligible sample size was 159,813, and the characteristics of the patients excluded were shown in Table 1. Among eligible patients 3.9% had their medications switched within 180 days of their initial prescriptions. More than one third of patients in our study were aged 70 years or older (34.0%); there were more women than men (54.8%) and most were not in receipt of public assistance (83.7%). Most of the patients attended in- and day-patient consultations (24.8%), specialist out-patient clinics (31.3%) or general out-patient clinics (34.9%) at their time of prescription. Greater proportions of patients lived in the New Territories (49.0%) and Kowloon (33.6%) than on Hong Kong Island (17.4%). Most were initially prescribed beta-blockers (42.5%) or CCBs (31.3%). Around 76% of patients did not have any medical conditions recorded which could influence the prescription choice of antihypertensive drug class.

**Table 1:** Characteristics of switchers and non-switchers.

	Switcher	Non-switcher	Overall	Excluded#
	N = 6163	N = 153650	N = 159,813	N = 63,474
**Age** *				
≤49	991 (2.6%)	36517 (97.4%)	37508 (23.8%)	24563 (38.7%)
50–59	1313 (3.9%)	32252 (96.1%)	33565 (21.0%)	9488 (15.0%)
60–69	1452 (4.2%)	32992 (95.8%)	34444 (21.6%)	8515 (13.4%)
≥70	2407 (4.4%)	51888 (95.6%)	54295 (34.0%)	20890 (32.9%)
Missing			1	18
**Gender** *				
Male	3197 (4.4%)	69010 (95.6%)	72207 (45.2%)	28718 (45.2%)
Female	2966 (3.4%)	84640 (96.6%)	87606 (54.8%)	34755 (54.8%)
Missing				1
**Payment status**				
Public assistance	1010 (4.1%)	23838 (95.9%)	24848 (15.5%)	8741 (14.0%)
Others	5117 (3.8%)	128602 (96.2%)	133719 (83.7%)	53892 (86.0%)
Missing			1246	841
**Service Type** *				
In-/Day- Patient	1867 (4.7%)	37758 (95.3%)	39625 (24.8%)	27589 (43.5%)
Specialist Out-Patient	1298 (2.6%)	48786 (97.4%)	50084 (31.3%)	16545 (26.1%)
Accident **&** Emergency	417 (4.3%)	9370 (95.7%)	9787 (6.1%)	7142 (11.3%)
General Out-Patient	2409 (4.3%)	53379 (95.7%)	55788 (34.9%)	10610 (16.7%)
Others	171 (3.8%)	4353 (96.2%)	4524 (2.8%)	1585 (2.5%)
Missing				3
**District** *				
Hong Kong	840 (3.0%)	26933 (97.0%)	27773 (17.4%)	11167 (17.6%)
Kowloon	2232 (4.2%)	51427 (95.8%)	53659 (33.6%)	21978 (34.6%)
New Territories	3091 (3.9%)	75290 (96.1%)	78381 (49.0%)	30329 (47.8%)
**Drug Class** *				
ACEI^1^	838 (4.5%)	17783 (95.5%)	18621 (11.7%)	4379 (6.9%)
alpha-Blockers	196 (2.1%)	9301 (97.9%)	9497 (5.9%)	3958 (6.2%)
beta-Blockers	2145 (3.2%)	65701 (96.8%)	67846 (42.5%)	30829 (48.6%)
CCB^2^	2192 (4.4%)	47780 (95.6%)	49972 (31.3%)	15604 (24.6%)
Thiazide	744 (5.6%)	12645 (94.4%)	13369 (8.4%)	4080 (6.4%)
Combined Fixed Dose	9 (12.2%)	65 (87.8%)	74 (0.0%)	33 (0.1%)
ARB	39 (9.0%)	395 (91.0%)	434 (0.3%)	200 (0.3%)
Combined	0	0	0	4391 (6.9%)
**Co-morbidity^∧^** *				
No	4060 (3.4%)	116789 (96.6%)	120849 (75.9%)	47955 (75.6%)
Yes	2103 (5.4%)	36861 (94.6%)	38964 (24.4%)	15519 (24.4%)
**Systolic blood pressure (mmHg)** * **(n = 108487)**	140.1 (13.8)	136.3 (13.3)	136.5 (13.3)	131.8 (18.5)
**Diastolic blood pressure (mmHg) (n = 108487)**	75.4 (8.9)	74.9 (8.6)	74.9 (8.6)	74.0 (10.1)

1 ACEI: Angiotensin converting enzyme inhibitors;

2 CCB: Calcium channel blockers.

# 27,238 visited the clinic setting only once, 4,391 were prescribed more than one antihypertensive agents, 31,845 stayed in the cohort for less than 180 days including die within the first 180 days or loss to follow up thereafter.

* Chi-square p-value for testing the association between switching status and the demographic variables <0.05.

### Factors Associated with Antihypertensive Drug Switching

Advanced age (>50 years) was significantly associated with medication switching (**Table 2**), but the association was not significant when both systolic and diastolic blood pressure levels were also included as covariates. Male subjects, residents in less urbanized regions (Kowloon and the New Territories) and those with medical conditions potentially confounding prescription choices were more likely to have their medication switched, whilst patients attending specialist out-patient clinics were less likely. Patients with higher SBP were more likely to be switchers. There is no difference in switching between in-patient and out-patient subjects.

**Table 2:** Factor associated with anti-hypertensive switching within 6 months after the first prescription.

	Crude OR	Adjusted OR	Adjusted OR	Adjusted OR with propensity score
			(with SBP and DBP)	(with SBP and DBP)
**Age**				
≤49	1.00	1.00	1.00	1.00
50–59	1.51 (1.38, 1.64)*	1.36 (1.25, 1.49)*	1.03 (0.93, 1.15)	0.91 (0.75, 1.09)
60–69	1.62 (1.49, 1.76)*	1.39 (1.27, 1.51)*	0.97 (0.87, 1.09)	0.80 (0.60, 1.06)
≥70	1.71 (1.59, 1.85)*	1.42 (1.31, 1.54)*	0.99 (0.88, 1.11)	0.79 (0.53, 1.16)
**Gender**				
Male	1.00	1.00	1.00	1.00
Female	0.76 (0.72, 0.80)*	0.71 (0.67, 0.75)*	0.65 (0.61, 0.70)*	0.37 (0.27, 0.51)*
**Payment status**				
Public assistance	1.07 (0.99, 1.14)	0.92 (0.85, 0.99)*	0.94 (0.87, 1.03)	0.93 (0.85, 1.02)
Others	1.00	1.00	1.00	1.00
**Service Type**				
In-/Day- Patient	1.25 (1.07, 1.47)*	1.17 (0.99, 1.38)	1.02 (0.85, 1.22)	1.14 (0.77, 1.69)
Specialist Out-Patient	0.67 (0.57, 0.79)*	0.74 (0.63, 0.87)*	0.71 (0.59, 0.85)*	0.94 (0.70, 1.27)
Accident & Emergency	1.13 (0.94, 1.36)	1.20 (0.99, 1.44)	1.16 (0.95, 1.42)	0.34 (0.12, 0.99)*
General Out-Patient	1.15 (0.98, 1.35)	1.11 (0.94, 1.30)	1.04 (0.87, 1.23)	0.65 (0.43, 0.99)*
Others	1.00	1.00	1.00	
**District**				
Hong Kong	1.00	1.00	1.00	1.00
Kowloon	1.40 (1.29, 1.52)*	1.48 (1.36, 1.61)*	1.70 (1.54, 1.88)*	1.83 (1.49, 2.26)*
New Territories	1.33 (1.23, 1.43)*	1.37 (1.27, 1.48)*	1.33 (1.21, 1.46)*	1.06 (0.81, 1.38)
**Drug Class**				
ACEI	1.00	1.00	1.00	1.00
alpha-Blockers	0.45 (0.38, 0.52)*	0.36 (0.30, 0.42)*	0.43 (0.35, 0.51)*	0.43 (0.36, 0.52)*
beta-Blockers	0.69 (0.64, 0.75)*	0.88 (0.80, 0.95)*	0.98 (0.88, 1.09)	0.98 (0.88, 1.09)
CCB	0.97 (0.90, 1.06)	0.97 (0.89, 1.05)	1.01 (0.90, 1.12)	1.01 (0.90, 1.12)
Thiazide	1.25 (1.13, 1.38)*	1.39 (1.25, 1.55)*	1.49 (1.31, 1.69)*	1.48 (1.31, 1.69)*
**Co-morbidity**				
(diagnosed within 180 days)				
No	1.00	1.00	1.00	1.00
Yes	1.64 (1.55, 1.73)*	1.50 (1.41, 1.59)*	1.48 (1.38, 1.59)*	1.48 (1.38, 1.59)*
**Systolic blood pressure (mmHg)**	1.02 (1.02, 1.02)*		1.02 (1.02, 1.02)*	1.02 (1.02, 1.02)*
**Diastolic blood pressure (mmHg)**	1.00 (1.00, 1.01)*		0.99 (0.99, 1.00)	0.99 (0.99, 1.00)*

* denotes p<0.05. ACEI: Angiotensin converting enzyme inhibitors; CCB: Calcium channel blockers.

### Association between Drug Class and Switching

**Figure 1:**
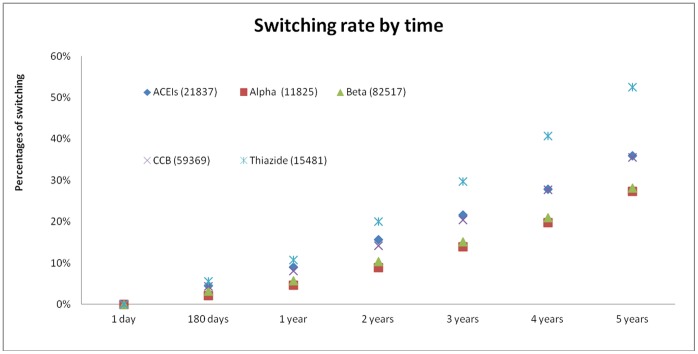
5-year cumulative switching rates of antihypertensive drugs according to medication class.

In multivariate regression models, thiazide diuretics were significantly more likely to be switched when compared to other major antihypertensive drug classes (adjusted odds ratio [AOR]: 1.49, 95% C.I. 1.31–1.69, p<0.001) (**Table 2**). When each patient was followed-up for 5 years, all antihypertensive drug classes showed a gradual escalation of their switching rates. The gradient of increase in cumulative rates of switching was most marked among patients prescribed thaizide diuretics (from 5.6% at 180 days to 52.5% at 5 years), whilst the increase in switching rates were similar among all other antihypertensive drug classes (5 year cumulative switching rates ranged between 27.3%–35.9%) (p<0.001) (**Figure 1**). The association between antihypertensive drug class and switching remained statistically similar when propensity score was included in the regression analysis.

## Discussion

### Major Findings

In this large cohort of 159,813 patients receiving a newly-initiated antihypertensive agent, 3.9% switched their medications within180 days of receiving their prescriptions. We found that patients first prescribed thiazide diuretics were significantly more likely to be switched, and a larger difference in switching was observed with time. Switching was more likely among male patients, residents in more rural areas and in-patients who had at least one medical condition that could potentially affect drug prescription choices. Higher levels of systolic blood pressure were associated with higher rates of switching.

### Relationship to Literature and Explanations

A retrospective cohort study using the health service database of Lombardia, Italy, evaluated residents on antihypertensive treatment aged 40–80 years during 1999–2002. The six month switching rate was 15% [17]. Similar database studies including pooled Italian, Swedish and Dutch cohorts reported a 9 month switching rate of 15.9% [30]. A historical analysis adopting the ambulatory claims of the Taiwan National Health Insurance found that the proportion of patients switching their antihypertensive agents ranged from 21–29% in a one-year follow-up period [16]. Our recent studies among Chinese hypertensive patients, including both new and follow-up clinic visitors in the years 2004–2007 [23], [31], showed that 5.7% had their medication switched which is compatible with the low switching rate in the current study. Hence, our present findings further strengthen our previous observation that antihypertensive medication switching is relatively low among ethnic Chinese patients when compared to Western countries.

In the majority of large scale administrative database studies conducted in Western countries, the hazard ratios of switching were highest for alpha-blockers, followed by CCBs and diuretics. ACEIs have been the drug class with the lowest switching rate after initiation [17]. Although these studies do not record the reasons for medication switching, a pharmacoepidemiological survey of 28,000 Italian physicians showed that adverse effects of antihypertensive medications were cited among the most common reasons. The most recognized adverse effects of thiazide diuretics have been development of incident diabetes and moderate potassium losses, which could in turn lead to increased blood pressure, salt sensitivity, higher rates of bone turnover and stroke [32].

The higher switching rate of thiazide diuretics in our study, implying a possibly greater incidence of adverse events in the Chinese, is a new finding. Male patients were especially liable to experience drug switching and the reasons are yet to be explored. This bears a significant practice implication as thiazide diuretics have been recommended as one of the first-line antihypertensive therapy for management of arterial hypertension according to the guidelines issued by the National Institute for Health and Clinical Excellence (2006) [33] and the seventh report of the Joint National Committee 7^th^ Report (2003) [34]. In Hong Kong a new guideline on management of arterial hypertension drawing on international experience has been developed and recommendations on the choice of first-line agents are similar [35].

Subjects aged 50 years or older (in models unadjusted for blood pressure levels), male patients, residents in more rural areas and those with at least one comorbid condition affecting antihypertensive drug choice were more likely to be switchers. The influence of age on the tendency to switch medications has been discussed in a recent study where switching rates of beta-blockers increased markedly from 14% for those aged <40 years to 30% for patients aged >75 years [16]. However, although most studies demonstrate that older patients adhere better to medication regimens than younger ones, these findings are inconsistent [36]–[39]. Conflicting data exist about whether male or female patients adhere better to antihypertensive treatments, as well on as the region of residence [36], [40].

### Strengths and Limitations

The large sample size, good dispensing practice of clinics and hospitals, completeness and capability of cross-referencing of the database when patients attend different clinics, and the accuracy of the prescription information represent the major strengths of this study. To the best of our knowledge this is the first evaluation of this scale which addressed the switching profiles of antihypertensive agents among Chinese patients in real clinical practice. However, there are limitations which should be mentioned. We do not know from our data the actual reasons why patients were switched. Changing clinics, patient-initiated requests for a different drug following pharmaceutical company promotional campaigns and marketing strategies targeted towards front-line physicians [16] may also play a role in antihypertensive drug switching in addition to the occurrence of adverse effects after initiation. Furthermore, some might argue that the availability of new medications in the classes of medications in the study period, physician bias on selection of prescriptions, the implementation of local guidelines, and inefficacy of the initial prescription might, and the presence of certain comorbidities like diabetes, renal diseases and other cardiovascular conditions also explain medication switching behaviors. Critics might also argue that in different clinics under the present study, the procedures and standard practice of measuring blood pressure might differ from each other. In addition, information on socioeconomic status is not usually available in large dataset studies, although this study consists of district of residence and receipt of public allowance as two proxy measures. Furthermore, this analysis also excluded a significant number of patients who visited the clinic only once, patients who died within 180 days after the initial prescription, stayed in the cohort for less than 180 days, or prescribed more than one antihypertensive agent. This might underestimate the real switching rate as they might be at higher risk of switching. Also, hypertensive patients in Hong Kong sometimes attend both the private and public sectors for antihypertensive medication refills, although more than 90% of hypertensive patients attended the public sector for management of hypertension. If switching occurred in the private sector this information would not be captured in the database used in this study, but all our research shows that older patients are unlikely to switch from public to private sectors for care of their non communicable diseases [41]. In addition, we do not take into account patients prescribed add-on pharmacotherapies within the follow-up period. However, this practice likely represents antihypertensive inefficacy instead of adverse effects as the study outcome, which should be explored in future evaluations. Finally, the initial antihypertensive agent prescription was not randomized despite our attempts for adjustment using propensity scores; hence one could not draw a definite conclusion on a cause-and-effect relationship between antihypertensive drug class and switching.

### Implications for Clinical Practice

This study provides clinicians and policy-makers with antihypertensive drug utilization profiles which are captured from real-life clinical practice. In addition, the low absolute rates of switching within 180 days after antihypertensive prescription should reassure physicians and their patients about the relatively low adverse reaction rates in the short-term. Reluctance to start antihypertensive drugs can lead to greater risks of cardiovascular morbidity and mortality [42], [43]. However, physicians should be vigilant when considering prescribing antihypertensives for patients who have the associated factors identified in this study as predisposing to the need for switching medications.

In summary, this study considers the frequency of switching new initiated antihypertensive agents in a large Chinese population. The higher odds of switching among thiazide users, especially among the male subjects, warrants further evaluation. Future studies should address the relationship between major antihypertensive drug classes and adverse effects in different ethnic groups.
